# Application of a novel modeling tool with multistressor functionality to support management of organic contaminants in the Baltic Sea

**DOI:** 10.1007/s13280-015-0668-2

**Published:** 2015-05-28

**Authors:** Emma Undeman, Bo G. Gustafsson, Christoph Humborg, Michael S. McLachlan

**Affiliations:** Department of Environmental Science and Analytical Chemistry, Stockholm University, 106 91 Stockholm, Sweden; Baltic Sea Center/Baltic Nest Institute, Stockholm University, 106 91 Stockholm, Sweden

**Keywords:** Baltic Sea, Organic pollutants, Eutrophication, Climate change, Chemicals management, Decision support

## Abstract

Organic contaminants constitute one of many stressors that deteriorate the ecological status of the Baltic Sea. When managing environmental problems in this marine environment, it may be necessary to consider the interactions between various stressors to ensure that averting one problem does not exacerbate another. A novel modeling tool, BALTSEM-POP, is presented here that simulates interactions between climate forcing, hydrodynamic conditions, and water exchange, biogeochemical cycling, and organic contaminant transport and fate in the Baltic Sea. We discuss opportunities to use the model to support different aspects of chemicals management. We exemplify these opportunities with a case study where two emission-reduction strategies for a chemical used in personal care products (decamethylcyclopentasiloxane) are evaluated, and where the confounding influence of future climate change and eutrophication on the impact of the emission-reduction strategies are assessed.

**Electronic supplementary material:**

The online version of this article (doi:10.1007/s13280-015-0668-2) contains supplementary material, which is available to authorized users.

## Introduction

Several environmental problems threaten the ecological status of the Baltic Sea and its catchment. Alongside eutrophication and loss of biodiversity, pollution by hazardous substances is recognized as one of the major stressors in this environment (HELCOM 2007). The need to consider the interactions between these and other stressors, such as climate change, when managing environmental problems has been highlighted (Schindler et al. 1995; Halpern et al. 2008; Curtin and Prellezo 2010; HELCOM 2013a). The multistressor approach is a major pillar in the concept of ecosystem-based management. As stated by HELCOM (2007): “The ecosystem approach is based on an integrated management of all human activities impacting on the marine environment and, based on best available scientific knowledge about the ecosystem and its dynamics, identifies and leads to actions improving the health of the marine ecosystem thus supporting sustainable use of ecosystem goods and services.”

The combination of various ongoing and future changes in environmental conditions, like global warming and nutrient emissions, will influence the release, transport, and fate of many organic chemicals used in society, either directly (e.g., higher temperatures may increase volatilization of organic pollutants from sea and land) or indirectly (e.g., increased organic carbon mass in aqueous and terrestrial systems may decrease volatilization of organic chemicals). In particular, the potential impact of climate change on contaminant levels has attained much attention in recent years (Macdonald et al. 2003; Schiedek et al. 2007; Noyes et al. 2009; Gouin et al. 2013). Modeling studies exploring interactions between climate change and contaminants have so far focused mainly on direct effects of climate change (changes in temperature, precipitation, wind speed, temperature-dependent degradation, ocean currents, sea–ice cover, etc.) on the global environmental fate of a number of legacy contaminants. The direct impact of these factors on environmental concentrations seldom exceeds a factor of two when comparing the predicted contaminant levels in the climate change scenario and the reference scenario (Gouin et al. 2013; Kong et al. 2013). However, additional feedbacks, such as changes in organic carbon cycling, terrestrial hydrology, and land use, which are more complex to model, are anticipated to have a more substantial impact on environmental contaminant transport and fate than the direct effects of climate change (Schiedek et al. 2007; Kallenborn et al. 2012; Gouin et al. 2013).

Re-emerging as an issue of scientific and management interest is the impact of organic carbon cycling on contaminant transport and fate (Borgå et al. 2010; Nizzetto et al. 2010, 2012; Berrojalbiz et al. 2011; Armitage and Wania 2013; Cabrerizo et al. 2013). Many organic chemicals sorb strongly to organic matter, making this matrix an important transport vector and sink for contaminants (Wania et al. 2000; Nizzetto et al. 2010). However, the organic carbon cycle itself is influenced by many external stressors. The strong increase in plankton biomass (e.g., during algal blooms) observed in the Baltic Sea during the latest decades is a consequence of increases in anthropogenic nutrient emissions, but it has also been influenced by other factors such as human-induced climate change and changes in food web structures (Meier et al. 2012).

Thus far, the large-scale impact of eutrophication on contaminant dynamics in the Baltic Sea has not been assessed, nor has the combined effect of future climate change and eutrophication on contaminant levels in this region. The consequences of multistressor pressure for chemicals management (e.g., will other stressors mitigate the impact of emission-reduction measures?) or for environmental monitoring (e.g., will other stressors cause time trends in contaminant levels that could be incorrectly attributed to changes in emissions?) have not been evaluated. One reason for this is the lack of appropriate modeling tools. The only multimedia fate model developed and parameterized for the entire Baltic Sea region is POPCYCLING Baltic (Wania et al. 2000), which has been used to study several organic contaminants (Breivik and Wania 2002; Mattila and Verta 2008; Armitage et al. 2009; Wiberg et al. 2009; Shatalov et al. 2012). This model, however, lacks a number of key features necessary to assess the complex interactions between multiple stressors and contaminant dynamics: (1) the organic carbon mass balance is not linked to external forcing like nutrient loads and meteorologic conditions; (2) water flow rates (e.g., inter- and intrabasin exchanges) are not estimated using hydrologic/physical models, but given as fixed yearly averages, thus excluding, e.g., seasonality and long-term climatic variations; (3) fixed average monthly values for meteorologic parameters such as air temperatures, wind speeds, and water temperatures (independent of air temperature) are applied and repeated each year of the simulation, and hence interannual variations and long-term trends in these parameters are not considered in the model.

Here, we present a recently developed modeling tool, BALTSEM-POP, which synthesizes knowledge about environmental processes and data from several scientific disciplines: meteorology, oceanography, biogeochemistry, ecology, and organic environmental chemistry. The model has the capacity to simultaneously simulate hydrologic circulation processes, heat fluxes, nutrient and carbon cycles, and organic contaminant transport and fate in the Baltic Sea as a function of meteorologic conditions and carbon/nutrient/contaminant loads from land and the atmosphere. The coupled hydrodynamic–biogeochemical submodel has been assembled using the best available knowledge concerning eutrophication in the Baltic Sea, and it has been employed in the eutrophication segment of the Baltic Sea Action Plan (BSAP, HELCOM 2007, 2013a). We discuss the utility of the BALTSEM-POP model for chemicals management in general and for facilitating the implementation of the segments of the HELCOM BSAP related to hazardous substances in particular. Finally, we apply the model in a model experiment to assess the influence of eutrophication and climate change on future environmental levels of decamethylcyclopentasiloxane (D5) in the Baltic Sea and illustrate how the model results can inform the management of this contaminant.

## The BALTSEM-POP model

BALTSEM-POP is a marine model that integrates hydrologic and biogeochemical cycles (e.g., nutrients and organic carbon) in the Baltic Sea with contaminant transport and transformation processes. It builds upon the BALTSEM model, which combines a hydrodynamic module (Gustafsson 2000a, b, 2003) and a biogeochemical module (Savchuk 2002; Savchuk et al. 2012), as well as a recently developed module for carbon cycling (Gustafsson et al. 2014). The model simulates water fluxes, salinity, temperature, concentrations of oxygen, silica, nitrogen, phosphorous, carbon, plankton, detritus, and organic pollutants in the Baltic Sea. It can be applied to neutral and (with some restrictions) ionic organic chemicals. The technical details of the BALTSEM-POP model have been presented elsewhere (Undeman et al. 2014).

### Utility of BALTSEM-POP for chemicals management

Chemicals management involves a vast range of activities, spanning from policy making to operational activities and monitoring. Ultimately, the goal is to reduce concentrations of harmful chemicals in the environment in the most cost-efficient way (Elofsson 2010). The multitude of interactions between meteorologic conditions, biogeochemical cycles, contaminant transport, and transformation processes complicate chemicals management in the Baltic Sea. Various environmental processes may enhance or counteract each other and impact chemical fate in ways that are difficult to foresee and/or quantify. With its multistressor functionality, BALTSEM-POP may be used in several ways to overcome these challenges and provide useful decision support.

One of the most important chemical-management activities is emission reduction. Evaluation of measures for emission reduction requires setting up a mass balance (also called a budget) that establishes the relative importance of various emission sources such as riverine inputs, direct emissions from point sources, atmospheric deposition, or re-emissions from contaminated sediments. This has been done for a number of organic contaminants in the Baltic Sea using multimedia fate models without multistressor functionality (Breivik and Wania 2002; Armitage et al. 2009; Wiberg et al. 2009). Mass balances can also be used to examine the completeness of emission inventories (Prevedouros et al. 2004; Shatalov et al. 2012). One of the most important uses of mass balance models is to compare the effects of different emission control policies (e.g., improved waste water treatment, regulations for industrial air abatement, banning of specific uses of chemicals) on contaminant concentrations in the environment. Hereby, two questions are frequently of interest: (1) what is the magnitude of any expected reductions in environmental concentrations?; (2) how long will it take to achieve this reduction? In contrast to other models, BALTSEM-POP can address these questions from a multistressor perspective. It can simulate the combined impact of measures to reduce eutrophication, global warming, and contaminant emissions to the Baltic Sea on organic contaminant concentrations in the ecosystem. It is hence possible to judge if management of other environmental problems in the Baltic Sea will counteract or enhance the outcome of various chemical-management strategies, and in that case, to what extent.

Another issue in chemicals management is the identification of particularly sensitive ecosystems. The Baltic Sea itself is considered a vulnerable region with its long residence time for water, the large population, and intense industrial and agricultural activities in its catchment, and its inherently low biodiversity due to the brackish water (Jansson and Dahlberg 1999). BALTSEM-POP can be used to identify which basins are particularly susceptible to elevated organic contaminant concentrations due to regional environmental conditions and water circulation patterns, and how this susceptibility may change depending on future management of eutrophication and global warming. BALTSEM-POP can also be applied to compare contributions to pollution of the individual basins from various regions/countries. The original BALTSEM model has previously been applied to identify region-specific contributions to the total nutrient load (nitrogen and phosphorous), allocate country-wise reduction targets for these elements, and to motivate differences in the economic burden (HELCOM 2013b).

Monitoring is also an important tool for the management of organic contaminants in the Baltic Sea. Management of organic pollutants in the Baltic Sea is in general hampered by lack of data, even for chemicals present on priority lists (Backer et al. 2010), and this makes monitoring and screening programs important activities in chemicals management. For instance, Baltic Sea-wide monitoring of hazardous substances is coordinated by HELCOM and is demanded by the EU Marine Strategy Framework Directive (MSFD). Several factors need to be considered when designing a monitoring program. For common monitoring parameters such as standard water-quality variables, statistical methods exist for optimizing the choice of sampling matrices, locations, timing, and frequency (Chapman et al. 1982; Droppo and Jaskot 1995; Dobbie and Negus 2013). However, organic contaminant monitoring is seldom data intensive, and consequently other tools are required to assess the available knowledge so that the monitoring program can be designed to provide the most useful information. BALTSEM-POP can be used to identify those environmental matrices in which the contaminant’s concentrations are likely to be the highest, ensuring that a matrix is selected in which the contaminant can be quantified. It can also evaluate how closely the temporal variation in the concentration in this environmental matrix is linked to the temporal variation in the emissions or in the exposure of endpoints of concern, which may be important considerations in assuring that the monitoring program delivers useful time trend information. The expected temporal and spatial variabilities of the concentration as a result of the variability in environmental conditions (e.g., variations in temperature and wind patterns, occurrence of algal blooms) can also be assessed so that sampling locations and time points can be chosen, which minimize unwanted impacts of this variability on the data. The rate and magnitude at which a change in an environmental parameter is reflected in the contaminant concentrations is chemical specific (Undeman et al. 2009), and depends also on the mode of emissions, i.e., whether contaminant emissions occur mainly to air, to water, to soil, or to sediment (Webster et al. 1998). Hence, these questions must be re-examined separately for each new contaminant. For the same reasons, the measured concentration of a contaminant will frequently be influenced by the environmental conditions prior to the sampling event. To understand and quantify this, models can be used to interpret monitoring data. The multistressor functionality of BALTSEM-POP creates new opportunities in this regard, for example, to assess whether a measured decline in contaminant concentration is attributable to interannual variations in climate, the timing/frequency of algal blooms, or reductions in chemical emissions.

Finally, an important use of multimedia models is to screen and prioritize among the thousands of chemicals with unknown environmental concentrations and effects that are used in society (Arnot and Mackay 2008; Brown and Wania 2008; Breivik et al. 2012). BALTSEM-POP may be used to predict and compare exposure levels specific for the Baltic marine environment for chemicals that are yet not measured, or even emitted.

### Support for implementation of the BSAP with respect to hazardous substances

The Helsinki Convention, signed in 1974 and expanded in 1992, is one of the most important multilateral actions to manage hazardous substances in the Baltic Sea (Selin and VanDeveer 2004; Backer et al. 2010). The HELCOM Baltic Sea Action Plan (BSAP) was adopted in 2007 and revised by a Ministerial Declaration in 2013 (HELCOM 2013a). The action plan is based on the so-called Ecosystem Approach, i.e., all stressors and their impacts on ecosystem functioning are considered simultaneously (Curtin and Prellezo 2010). It includes specific actions for achieving a number of ecological objectives (e.g., “Concentrations of hazardous substances close to natural levels” and “Natural level of algal blooms”). The BSAP also defines initial targets and indicators for measuring progress toward the ultimate goal, namely, a Baltic Sea in Good Environmental Status by 2021. While targets and quantitative emission-reduction goals are well defined for nutrients, the segment on hazardous substances in the BSAP is more focused on gathering information about selected substances (Backer et al. 2010). There are several ways in which BALTSEM-POP can support the implementation of the BSAP for organic contaminants. Several applications are discussed in the following.

The BSAP calls for development, identification, and evaluation of measures to reduce emissions of the 11 priority substances/substance groups identified by HELCOM. This has been done in the COHIBA project (http://www.cohiba-project.net/), and the effectiveness of each identified measure has been assessed by calculating the fraction reduction of the total load to the Baltic Sea and the cost per kg of reduced chemical emissions (Menger-Krug et al. 2011; HELCOM 2013a). BALTSEM-POP may be applied to assess how region-specific reductions in air concentrations and river loads are propagated in the different basins of the Baltic Sea. The model can hence enable comparisons not just of reductions in total loads to this region, but also of exposure levels in water and sediments in the various Baltic basins. Furthermore, BALTSEM-POP would provide information on the time delay associated with the expected impact of each identified measure. Another application is to assess the influence of other stressors such as climate change and eutrophication on the expected impact of each identified measure, in accordance with the Ecosystem Approach anchored in the BSAP. For example, the explicit BSAP activities to develop measures to control large-scale industrial sources of dioxins and to prevent pharmaceuticals from reaching the Baltic Sea (HELCOM 2013a) could be supported in all three of these ways using BALTSEM-POP.

The BSAP calls for making use of substance-specific information generated by REACH, the WFD, and the MSFD. In BALTSEM-POP, basic data on physical chemical properties, production volumes, emission factors, and use data (converted to emission scenarios for air and rivers) may be transformed into metrics valuable in chemicals risk assessment, such as predicted environmental concentrations (PECs) that may be compared to toxicological thresholds or thresholds for good environmental status. Such basic chemical information can also be used by BALTSEM-POP to screen for new candidate priority substances for inclusion in the Stockholm Convention and Aarhus Protocol on POPs as specified in the BSAP, e.g., by doing a comparative evaluation of the persistence of a wide range of chemicals in the Baltic Sea.

The Monitoring and Assessment Strategy adopted in the BSAP (HELCOM 2013a) addresses the need to link various anthropogenic pressures to the current state of the sea, and to provide guidance for future responses to changes in the system (e.g., in the Holistic Assessments of Ecosystem Health produced by HELCOM). In addition to the abovementioned possibilities to use BALTSEM-POP for optimization of monitoring program design, this tool is useful for synthesizing the emission inventories and field data collected by HELCOM, and making projections of future changes in environmental chemical pollution due to changing emissions of chemicals, emissions of nutrients, and climate.

## Materials and methods

To illustrate the capabilities of BALTSEM-POP, a case study is presented in which the model is used to evaluate and compare two emission control options for decamethylcyclopentasiloxane (D5) and to assess how future climate change and eutrophication will impact the outcome of the emission-reduction efforts. D5 belongs to a group of emerging pollutants, the cyclic volatile methylsiloxanes (cVMS). D5 is a high production volume industrial chemical used in silicon polymer production and as a carrier in personal care products (e.g., shampoo, deodorants). The use of personal care products containing D5 is the largest source of its emissions to the environment. D5 possesses unusual physical chemical properties (high volatility, high hydrophobicity, susceptibility to hydrolysis in water, persistence when sorbed to particles), and is classified as a suspected vPvB (very persistent, very bioaccumulative) chemical in the EU risk assessment (Brooke et al. 2009).

The BALTSEM-POP model was run for D5 using different emissions, eutrophication, and climate scenarios. The scenarios and the physical–chemical properties of D5 used in the simulations are described in detail in the Supplementary Material. Two emission control scenarios were considered: (1) 90 % reduction of D5 concentrations in the atmosphere, for instance by banning its use in personal care products applied directly to the skin (e.g., deodorants), and (2) 90 % reduction of river loads of D5, for instance by restriction/banning its use in personal care products applied in the shower (e.g., shampoo). Future concentrations in the Baltic Sea were simulated using various combinations of scenarios for D5 emissions, climate conditions and nutrient loads. First, the most efficient measure to reduce D5 concentrations in water and sediment was identified by comparing the future D5 concentrations in these matrices under each of the two D5 emissions scenarios, assuming current climate conditions (“random weather” RW) and nutrient loads (“constant loads” CL). Then, for the emissions scenario giving the greatest reduction, the influences of changing climate and trophic status on the future D5 concentrations were explored by comparing simulations made using combinations of different scenarios for eutrophication (increasing eutrophication, i.e., constant loads of nutrients at today’s levels [CL], and reduced eutrophication as a result of implementation of the BSAP for nutrients [BSAP]) and climate (no climate change [RW] and severe climate change [a1b]). In short, the a1b scenario results in on average 7 % higher wind speed, 60 % higher air temperatures, and 20 % more precipitation in the entire Baltic Sea compared to the RW scenario. A summary of the scenarios is presented in Tables S1 and S2 in the Supplementary Material. Note that in this model experiment, hypothetical but realistic emission scenarios were constructed (see description in Supplementary Material and Fig. S2). It is beyond the scope of this study to make a full emission inventory for D5 in the Baltic Sea region.

## Results and discussion

**Fig. 1:**
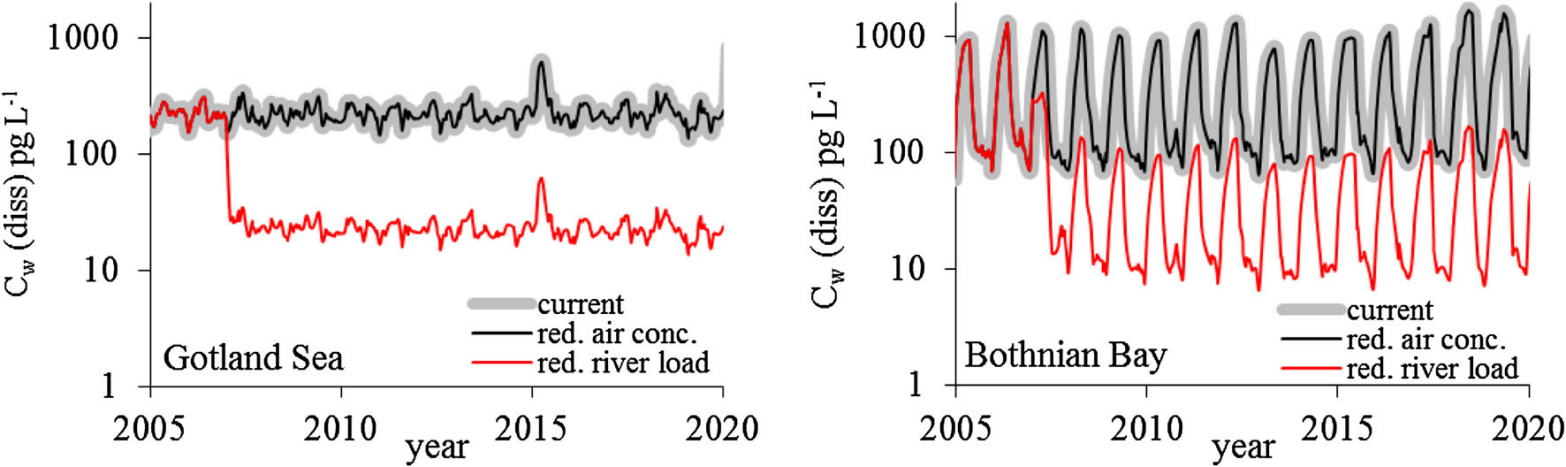
Predicted surface water concentrations (dissolved, in pg L^−1^ bulk water at 10-m depth) in the Gotland Sea and Bothnian Bay with emissions either constant at current levels, emissions via rivers reduced (year 2006) by 90 %, or air concentrations reduced by 90 % (labeled current, red. river load, and red. air conc. in the legend, respectively). The forcing scenario RWCL was used, i.e., the nutrient loads were fixed at a level representing the average between 1997 and 2003 (constant load, CL), and the climate scenario represents a random weather (RW) similar to today’s conditions (no further global warming)

Figure 1 shows the effectiveness of the two emission-reduction strategies at reducing dissolved surface water concentrations of D5 (*C*_W_, pg L^−1^ on a bulk water basis) in two Baltic basins (the Gotland Sea and Bothnian Bay, see Fig. S1 in Supplementary Material). Corresponding results for the Fehmarn Belt are shown in the Supplementary Material (Fig. S3). The simulations show that reducing air concentrations by 90 % in year 2006 has practically no influence on the concentrations in surface water in either basin. This is because the chemical potential of D5 is much greater in the seawater than in the air, resulting in a very strong diffusion gradient from the sea to the atmosphere. Reducing river loads by 90 % in 2006 lowers the concentrations in surface water by 90 % in both basins. Restrictions in the D5 uses that result in emissions to waste water (e.g., in shampoo) are hence most effective at reducing environmental levels.

**Fig. 2:**
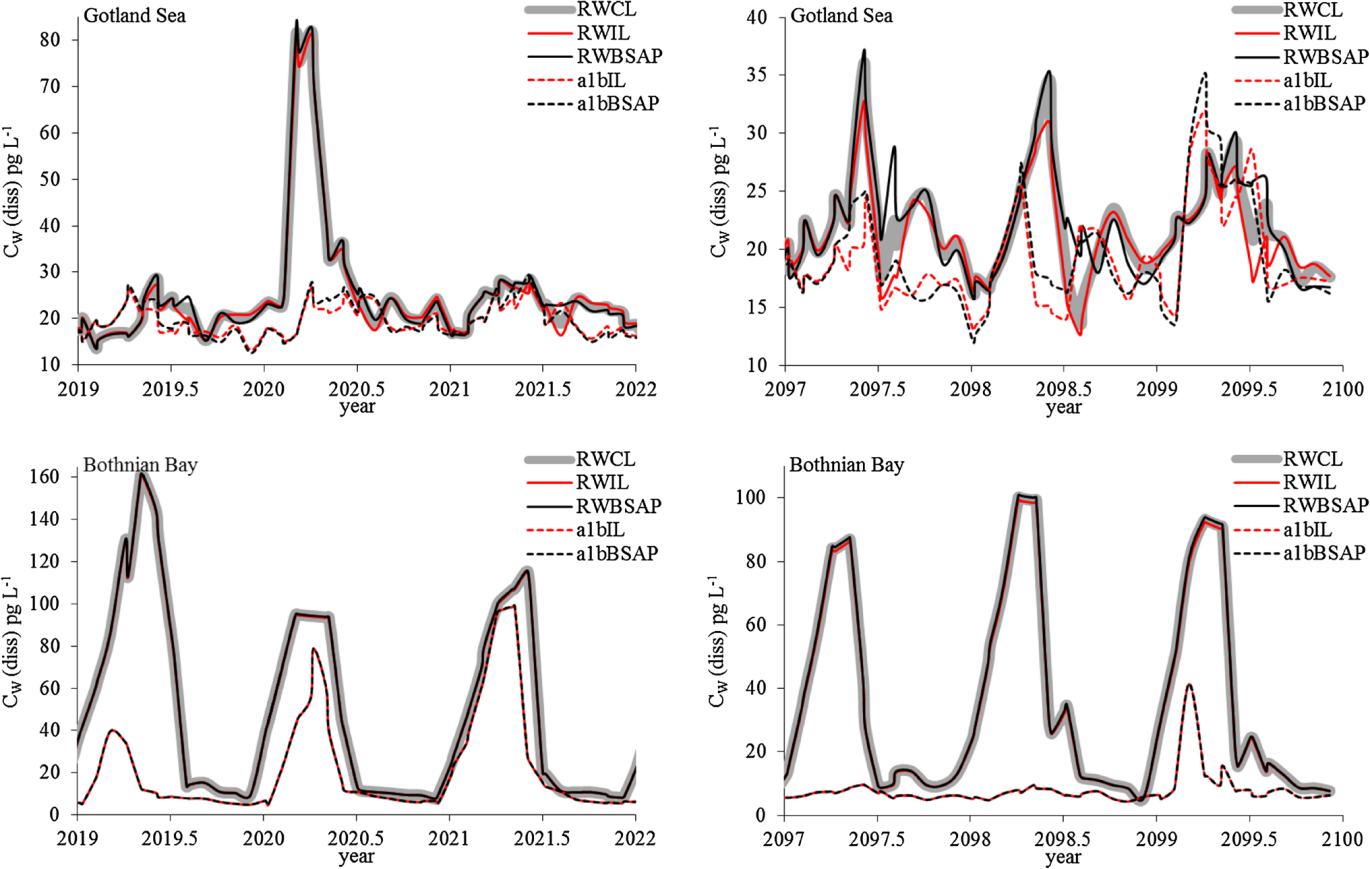
Predicted surface water concentrations (dissolved, in pg L^−1^ bulk water at 10-m depth) in the Gotland Sea and Bothnian Bay during two time periods (2019–2022 and 2097–2100) calculated using five different scenarios for climate change and nutrient loads. See also Fig. S5 in the Supplementary material

Additional simulations were performed to assess possible impacts of future changes in climate and trophic status in the Baltic Sea on the efficiency of the emission control. Figure 2 shows how climate change and nutrient loads impact the concentration of freely dissolved D5 in the Gotland Sea and Bothnian Bay (results for Fehmarn Belt are shown in Fig. S4).

The results indicate that for D5, future changes in nutrient loads are likely to have a minor impact on the concentrations in water. The scenarios with random weather combined with increasing load of nutrients (RWIL) and with the BSAP for nutrients implemented (RWBSAP) yield a very similar predicted water concentration *C*_W_ around the year 2021 (when the BSAP goals are anticipated to be fulfilled). Eighty years later the effects of the increasing/decreasing nutrient loads are more prominent. For instance, the detritus biomass in the Gotland Sea is predicted to be on average ca. 3.4 times higher between years 2090 and 2100 in the RWIL than in the RWBSAP scenario, compared with 1.7 times higher between 2015 and 2025. At this time, the predicted *C*_W_ in the Gotland Sea is on average ca. 7 % higher for the RWBSAP-scenario compared to the RWIL-scenario. No eutrophication is predicted to occur in the more oligotrophic Bothnian Bay; the carbon mass increases by less than ca. 20 % in the RWIL scenario and the D5 concentrations are not significantly impacted by the increased nutrient load.

The impact of climate change on *C*_W_ (scenarios a1bIL and a1bBSAP) is stronger. In the Gotland Sea, *C*_W_ is generally lower with the a1b climate scenario, but there are also periods when *C*_W_ is higher than that with the RW climate scenario. The randomness of weather conditions, e.g., unusually stormy or warm years in either scenario, has a stronger influence on *C*_W_ than the long-term trends in climate. However, the 10-year average *C*_W_s calculated between 2010 and 2100 (Fig. S5) shows that the climate change simulated using scenario a1b lowers the water concentration by ca. 20 % compared to current climate conditions simulated using the scenario RW.

In the Bothnian Bay, however, climate change lowers the 10-year average *C*_W_ of D5 in surface water by ca. 45–80 %, with the difference between the scenarios increasing over time. The lower panels in Fig. 2 also display a considerably stronger impact of climate change during 2097–2100 compared to the years 2019–2022; *C*_W_ is up to 15 times lower in the warmer climate. The explanation for these results is the predicted reduction in sea–ice cover due to higher temperatures by the end of the century. As shown in Figs. 1 and 2, *C*_W_ shows strong seasonal variations in the Bothnian Bay (and in the Gotland Sea during 1 year, 2020), with a strong increase during winter followed by a decrease during spring. This corresponds to a sharp decrease in the loss via volatilization from the surface water to the atmosphere during winter when sea–ice covers this basin (Fig. S6). Since volatilization is the major loss process for D5 in surface water, this results in a fast increase in *C*_W_. In the a1b climate scenario, little-to-no sea–ice forms in the Bothnian Bay during 2097–2100, and consequently no winter peak in *C*_W_ occurs (Fig. 2, lower right panel). Hence, climate change can be expected to strongly enhance the effect of emission control measures for D5 in the northern Baltic Sea.

## Conclusion

The novel model, BALTSEM-POP, can support chemicals management in the Baltic Sea in several ways. It can be applied to compare the efficiencies of alternative emission-reduction measures; to compare the sensitivities of the different basins to pollution; to allocate region-specific emission-reduction goals (similar to what has previously been done for nutrients); to contribute to the understanding of organic contaminants’ major emission sources and transport routes in the marine environment; to optimize monitoring programs and help us interpret monitoring data; and to screen for emerging contaminants using Baltic Sea-specific selection criteria. For all these applications, the potential multistressor impacts from eutrophication and climate change may be considered. The case study for D5 exemplifies how BALTSEM-POP can be used in a simple way to support chemicals management in the Baltic Sea. In summary, this model experiment has provided several pieces of information of value for managing D5 in the Baltic Sea. To reduce levels of D5 in the Baltic Sea, the best strategy is to reduce D5 emissions to water. The areas of the Baltic Sea experiencing the highest D5 exposure will be those that combine high emissions to water with seasonal ice cover, and in these areas the highest concentrations will occur at the end of the winter. From an ecosystem-based management perspective, future trophic status of the Baltic Sea will not impact concentrations of D5 in the water significantly, whereas climate change can be important in regions that currently have seasonal ice-cover.

## Future research

The development of the BALTSEM models is an ongoing activity at the Stockholm University Baltic Sea Center/Baltic Nest Institute. Currently, BALTSEM-POP is a purely marine model, with chemical concentrations in air and river loads given as external forcing. A catchment module is, however, currently under development to enable the model to simulate the entire transport chain from land-based emissions to air, fresh water, or soil to the marine environment. With these features, the model will be better suited for evaluating, e.g., emission controls for chemicals released in the catchment/terrestrial system, such as pharmaceuticals and pesticides. The model can also be improved by implementing additional algorithms for ionizing chemicals and metals, two chemical classes that are currently outside the BALTSEM-POP range of applicability. We also plan to incorporate a food web model into the modeling platform to allow for assessment of the exposure of higher trophic level organisms to organic contaminants. Finally, linking BALTSEM-POP to economic models would provide a tool that integrates cost estimations in the evaluation and optimization of various chemical-management strategies. The ultimate goal of these model development and application activities is to provide support for an objective and systematic strategy for management of the thousands of chemicals present in the Baltic Sea.

## Electronic supplementary material

Supplementary material 1 (1249 kb)
