# Supplementary material for: Application of a novel modeling tool with multistressor functionality to support management of organic contaminants in the Baltic Sea
Source: Ambio. 2015 May 28;44(Suppl 3):498–506. doi: 10.1007/s13280-015-0668-2 (PMC4447700; doi:10.1007/s13280-015-0668-2)
Supplement: Supplementary file 1 — Supplementary material 1 (1249 kb) [file 13280_2015_668_MOESM1_ESM.pdf]

AMBIO

Electronic Supplementary Material

This supplementary material has not been copy edited by the publisher or the editorial office.

Title: **Application of a novel modeling tool with multi-stressor functionality to support management of organic contaminants in the Baltic Sea**

Authors: Emma Undeman, Bo Gustafsson, Christoph Humborg, and Michael McLachlan

BALTSEM-POP

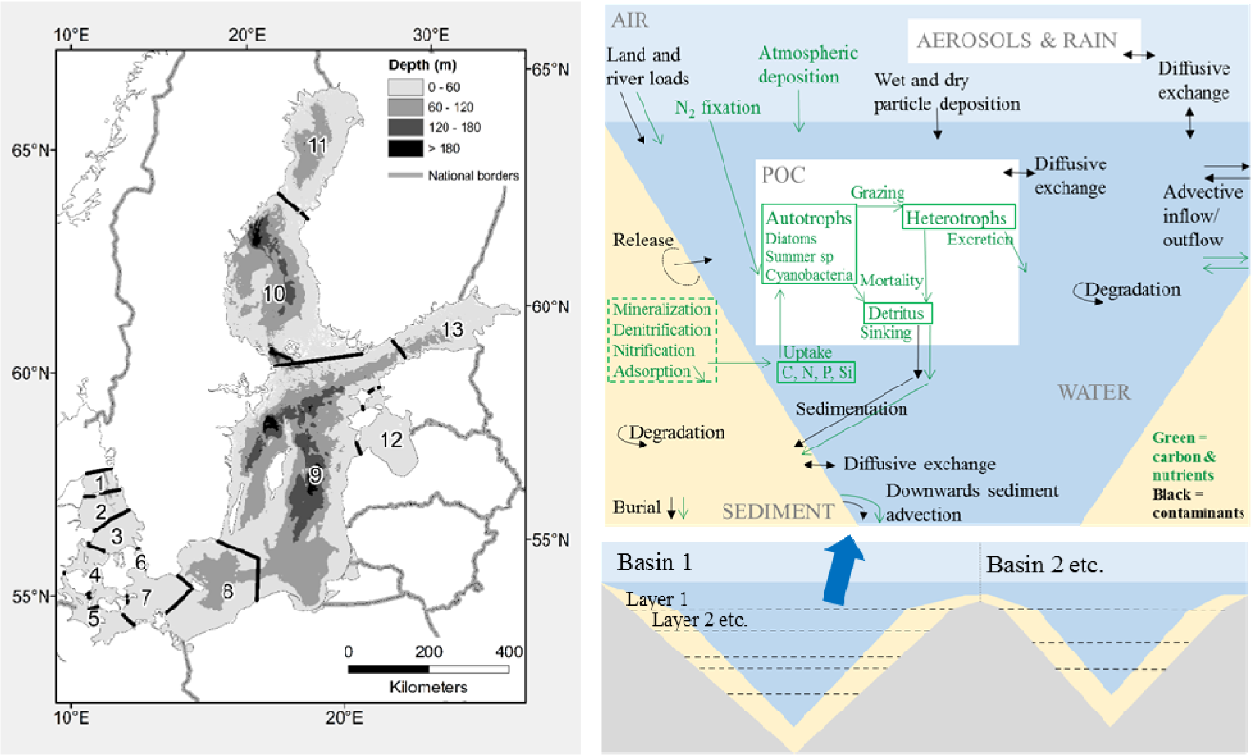

Figure S1. *Overview of the BALTSEM-POP model. Left panel: The Baltic Sea divided into thirteen sub-basins (adopted from Savchuk et al., 2012). The Baltic Sea is divided into thirteen sub-basins: 1. Northern Kattegat (NK), 2. Central Kattegat (CK), 3. Southern Kattegat (SK), 4. Samsø Belt (SB), 5. Fehmarn Belt (FB), 6. Öresund (OS), 7. Arkona basin (AR), 8. Bornholm basin (BN), 9. Gotland Sea (GS), 10. Bothnian Sea (BS), 11. Bothnian Bay (BB), 12. Gulf of Riga (GR), 13. Gulf of Finland (GF). Right panels: Overview of processes for nutrient, carbon and contaminant transport and transformation included in surface water layers in all BALTSEM-POP basins. Deeper water layers include similar processes except exchange with the atmosphere. Carbon/contaminant interactions considered are diffusive exchange between water and particulate organic carbon (POC) and diffusive exchange between overlaying water and sediment. The absorbed contaminants are transported with the movement of these matrices (e.g. via sedimentation of particles, burial of sediment etc.)*

Forcing scenarios and the physical-chemical properties of decamethylcyclopentasiloxane

Forcing scenario for D5 emissions

A hypothetical, but reasonable scenario for air concentrations and river loads was assembled for decamethylcyclopentasiloxane (D5). The air concentration scenario was derived using measured air concentrations from a rural site in Southern Sweden (McLachlan et al., 2010). The measurements ranged from 20th of January to 15th of June, and were mirrored over the second half of the year (data from first and last sampling period was repeated to cover the full year, see Fig. S2). Reaction with OH radicals in the atmosphere is the major loss process for D5 in the atmosphere. Generally higher concentrations of these radicals in summer explains the seasonality of the concentrations (McLachlan et al., 2010). The modeling work in that paper showed only small spatial differences in D5 concentrations in the Baltic Sea region.

Total river loads (mg day^{-1}, see Fig. S2) to each BALTSEM-POP basin were estimated from per capita use data, removal efficiency in sewage treatment plants and average residence time in the watersheds, assuming steady state concentrations in all rivers (i.e. total input = total output):

Steady state river concentration (Eq S1):

Assume no change in river concentration with time:

Input from STPs = In river dissipation + Output to Baltic Sea

This equals (units below):

$Pop \times UsePerCapita \times STPremov = DissRate \times Volume \times C_{river} + StreamFlow \times C_{river}$

pers mg/pers fraction 1/day m^3 mg/m^3 m^3/day mg/m^3

$Pop \times UsePerCapita \times STPremov = C_{river}\ (DissRate \times Volume + StreamFlow)$

$C_{river} = Pop \times UsePerCapita \times STPremov\ /\ (DissRate \times Volume + StreamFlow)$

where

$Volume = StreamFlow \times WatResTime$

m^3 m^3/day days

Then:

$C_{river} = Pop \times UsePerCapita \times STPremov\ /\ StreamFlow\ (DissRate \times WatResTime + 1)$

Multiply both sides with StreamFlow to get River load with units mg/day:

River load = $(Pop \times UsePerCapita \times STPremov)\ /\ (DissRate \times WatResTime + 1)$

Where *Pop* (pers) is the total population in all watersheds connected to the BALTSEM-POP basin, *UsePerCapita* (11.6 mg pers^{-1} day^{-1} (Price et al., 2010)) is the daily emission to STPs per person, *STPremov* (ca. 0.95,(Price et al., 2010)) is the fraction removed by sewage treatment, *WatResTime* is the average water residence time (days) in all watersheds connected to the BALTSEM-POP basin and *DissRate* (0.4 day^{-1} (Price et al., 2010)) is the dissipation rate (due to hydrolysis, volatilization and sedimentation in the rivers). Since BALTSEM-POP

does not include a hydrological watershed model, the average river water residence time was estimated using a population weighted average distance from all households in a BALTSEM-POP basin catchment to the sea (i.e. the length of the river stretch) and an average stream velocity of the rivers in the catchment. The distance to sea was calculated using ArcGIS. The approximate average stream velocity (m s^{-1}) was estimated from the population weighted average stream order in each catchment (i.e. the stream order of rivers close to large populations were given more weight) and stream order specific velocities derived by (Humborg et al., 2010). The calculated residence times (yearly averages, in days) were:

Basin 1	Basin 2	Basin 3	Basin 4	Basin 5	Basin 6	Basin 7	Basin 8	Basin 9	Basin 10	Basin 11	Basin 12	Basin 13
1.5	0.6	0.6	0.4	0.5	0.2	0.3	3.9	4.2	1.0	0.9	2.8	1.0

The river loads (tonnes $month^{-1}$) were transformed to river water concentrations using runoff with monthly resolution (m^3 $month^{-1}$).

Initial concentrations in marine water and sediment were set to zero. The air concentration and river load scenarios were repeated each year of the simulation time period (1985-2100).

The emission scenarios entailed a step reduction in emissions to air or water by 90% at the end of 2006. This was simulated by lowering the air concentrations and river loads by 90%, respectively. Most of D5 release to the environment originates from personal care products. Almost all of D5 applied to skin is emitted to air while most of the D5 used in hair care products in the shower goes down the drain (Brooke et al., 2009). Hence eliminating D5 use in personal care products applied to the skin and used in the shower could plausibly lead to these reductions in emissions to air and water, respectively.

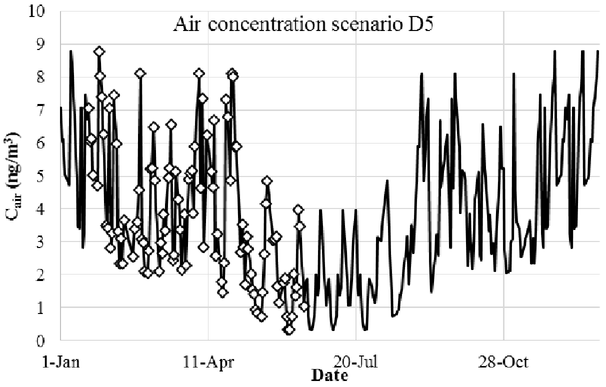

River loads (tonnes/month)	
Basin 1	0.22
Basin 2	0.24
Basin 3	0.22
Basin 4	0.26
Basin 5	0.43
Basin 6	0.32
Basin 7	0.17
Basin 8	2.38
Basin 9	4.41
Basin 10	0.52
Basin 11	0.26
Basin 12	0.61
Basin 13	2.60

Figure S2. *Air concentration scenario used in model simulations for D5 all years and basins (solid black line) and measurements from Southern Sweden in 2009 (diamonds) (McLachlan et al., 2010). The table shows monthly river loads used for each basin.*

Forcing scenario for climate and nutrient loads

Five scenarios were used for various combinations of future climate and nutrient loads:

- Random Weather and Constant (current day) nutrient Loads (**RWCL**)
- Random Weather Increasing nutrient Loads (**RWIL**)
- Random Weather and Baltic Sea Action Plan for nutrients implemented (**RWBSAP**)
- Climate scenario corresponding to emission scenario A1B and Increasing nutrient Loads (**a1bIL**)
- Climate scenario corresponding to emission scenario A1B and Baltic Sea Action Plan for nutrients implemented (**a1bBSAP**)

The three nutrient load scenarios have seasonal variation, but are otherwise constant with time. Thus, nutrient loads are completely invariant to climate change scenarios. The total nitrogen (TN) and total phosphorus (TP) loads are given in Table S1, but the actual loads are split between organic and inorganic fractions from atmospheric deposition, rivers and coastal point sources. The current day and Baltic Sea Action Plan (BSAP) loads are obtained from HELCOM (2013), while the increasing loads scenario represent a case of intensified agriculture as described by Humborg et al. (2007).

In addition to nutrient and contaminant loads, the forcing needed to run BALTSEM comprise of atmospheric forcing variables (temperature, wind, humidity, precipitation, cloudiness, air pressure and pCO2), daily averaged sea level observations from southern Kattegat and of S, T, and concentrations of other constituents at the boundary to Skagerrak and river runoff with associated concentrations of DIC and DOC.

The Random Weather scenario, represent a climate without long-term trends, although with variation. This scenario is based on a synthetic meteorological forcing data set. There are some basic requirements on the synthetic forcing data sets, they need to be spatially consistent for different parts of the Baltic and covariance's between variables needs to be maintained. BALTSEM does not explicitly simulate inertia (waves) and therefore temporal jumps in forcing do not have critical consequences for the results except that unrealistic jumps in the sea level time-series must be avoided since such would cause unrealistic inflow events to the Baltic proper. A simple method of random selection is used to create new forcing time series. First, the original forcing time series are sliced at each instant of zero sea-level in Kattegat. The time of year is noted for each slice. Then, new long time series of forcing are created by random selection of the slices, with only constrain that the slice should originate from the same time of the year (within a three month interval). To get best possible representation of natural variability the reconstructed forcing 1850-2006 (Gustafsson et al, 2012) is used to construct the statistical forcing. Concentrations in Skagerrak are assumed to be constant, following the average annual cycle obtained from the data from approx. 1980-2006.

The climate change scenario was derived in the ECOSUPPORT project and was produced by downscaling an ECHAM5 global projection of the A1B emission scenario with the RCAO regional climate model with 25 km resolution (Meier et al., 2011). River runoff was estimated from a statistical relationship between observed river runoff and the net precipitation minus evapotranspiration of the RCAO and sea levels in Kattegat was estimated using a relation between sea levels and atmospheric pressure fields (Meier et al., 2011, 2012). A time-series of pCO2 representing the emission scenario is also applied. A detailed description of the climate

change scenario is available in the abovementioned publications. In short, the A1B greenhouse gas emission scenario results in on average 7% higher wind speed (W), 60% higher air temperatures (T) and 20% more precipitation (P) in the entire Baltic Sea compared to the random weather (RW) scenario (in the Gotland Sea: W 10%, T 50%, P 20%, Bothnian Bay: W 25%, T 150%, P 30%, Fehmarn Belt: W 5%, T 35%, P 25% higher than the RW scenario for these individual basins).

***Table S1**: Loads of nitrogen (TN) and phosphorus (TP) in the scenarios. Constant loads and BSAP are obtained from HELCOM (2013) and increasing loads are calculated from the scenario of Humborg et al. (2007). Loads are given in t yr^{-1}.*

	**Constant loads**		**BSAP**		**Increasing loads**	
	TN	TP	TN	TP	TN	TP
Basin 1-3	78761	1687	74000	1687	78761	1687
Basin 4-6	65472	1601	65472	1601	65472	1601
Basin 7-9	423901	18320	325000	7360	655476	29521
Basin 10	79372	2773	79372	2773	79372	2773
Basin 11	57622	2675	57622	2675	57622	2675
Basin 12	88418	2328	88418	2020	132913	5133
Basin 13	116252	7509	101800	3600	178204	10314
Sum	*909799*	*36894*	*791685*	*21716*	*1247820*	*53704*

***Table S2.** Summary of scenarios used in the simulations.*

**Scenario name**		**Weather**	**Nutrient Load**	**Reduction of D5 emissions to water**	**Reduction of D5 emissions to air**
**current**	**current** weather conditions and nutrient load	random (today's condition, no further global warming)	constant (average between 1997-2003)	0%	0%
**red. air conc.**	current conditions and **red**uced **air** D5 **conc**entrations	random (today's condition, no further global warming)	constant (average between 1997-2003)	0%	90%
**red. river load**	current conditions and **red**uced **river** D5 **load**	random (today's condition, no further global warming)	constant (average between 1997-2003)	90%	0%
**RWCL**	**r**andom **w**eather, **c**onstant **l**oad	random (today's condition, no further global warming)	constant (average between 1997-2003)	90%	0%
**RWIL**	**r**andom **w**eather, **i**ncreasing **l**oad	random (today's condition, no further global warming)	increasing load	90%	0%
**RWBSAP**	**r**andom **w**eather, **BSAP** implemented	random (today's condition, no further global warming)	BSAP implemented (decreasing load)	90%	0%
**a1bIL**	climate scenario **a1b**, **i**ncreasing **l**oad	GHG-emission scenario a1b (global warming)	increasing load	90%	0%
**a1bBSAP**	climate scenario **a1b**, **BSAP** implemented	GHG-emission scenario a1b (global warming)	BSAP implemented (decreasing load)	90%	0%

Physical chemical properties of D5

Table S3. *Physical-chemical properties of D5 used in model simulations. Properties for D5 taken from (Brooke et al., 2009; Whelan, 2013; Xu and Kropscott, 2012a, b)*

Property	Abbreviation	Unit	D5	Ref
Molecular weight	MW	$g\ mol^{-1}$	371	
oct-water partition coefficient	$\log K_{OW}$	$m^3 m^{-3}$	8.07	*a*
air-water partition coefficient	$\log K_{AW}$	$m^3 m^{-3}$	3.13	*a*
oct-air partition coefficient	$\log K_{OA}$	$m^3 m^{-3}$	4.95	*b*
heat of phase transfer oct-water	U_{OW}	$J\ mol^{-1}$	29000	*c*
heat of phase transfer air-water	U_{AW}	$J\ mol^{-1}$	80400	*c*
heat of phase transfer oct-air	U_{OA}	$J\ mol^{-1}$	-48700	*b*
degradation half life water	$t½_{W}$	year	calculated (ca 1 at pH 8, 4°C)	
activation energy water	AE_{W}	$J\ mol^{-1}$	see below	
degradation half life sediment	$t½_{sed}$	year	calculated (ca 1400 at pH 8, 4°C)	
activation energy sediment	AE_{sed}	$J\ mol^{-1}$	see below	
**Additional properties used for calculation of degradation half lives**				
organic carbon-water part. coeff.	$\log K_{OC}$	$m^3 m^{-3}$	5.17	*d*
rate constant for the hydronium ion-catalysed reaction (25°C)	k_{H3O}	$L\ mol^{-1}\ h^{-1}$	742	*d*
rate constant for the hydroxide ion-catalysed reaction (25°C)	k_{OH}	$L\ mol^{-1}\ h^{-1}$	3200	*d*
activation energy for acid-catalysed reaction	AE_{H3O}	$J\ mol^{-1}$	59400	*d*
activation energy for base-catalysed reaction	AE_{OH}	$J\ mol^{-1}$	87200	*d*

a = (Xu and Kropscott, 2012a), *b* = (Xu and Kropscott, 2012b), *c* = (Whelan, 2013), *d* = (Brooke et al., 2009)

Calculation of hydrolysis rate constants

The degradation of D5 is strongly temperature and pH dependent (Brooke et al., 2009). BALTSEM-POP was therefore slightly modified to calculate the degradation rates in water and sediment as a function of these parameters using measured rate constants for the hydronium ion-catalyzed and acid-catalyzed hydrolysis at a reference temperature (25°C) (Brooke et al., 2009).

The two reference rate constants (k_{H3O} and k_{OH} with units $L\ mol^{-1}\ h^{-1}$) were temperature corrected using the Arrhenius equation:

$$k_{deg}(T_{env}) = k_{deg}(T_{ref}) \exp(AE/R \times (1/T_{ref} - 1/T_{env}))$$

where AE is activation energy for acid-catalysed or base-catalysed reaction and T is the environmental or reference temperature (K). The temperature corrected rate constants were

multiplied by the hydronium and hydroxide ion concentrations (mol L^{-1}) and summed to estimate an overall degradation rate constant (in h^{-1}):

$$k_{deg} = k_{H3O}\,[H_3O^+] + k_{OH}\,[OH^-]$$

Following the method described by Whelan (2013), it was assumed that hydrolysis is the only degradation process, and that in sediments this reaction takes place only for freely dissolved molecules in the pore water. The total hydrolysis rate constant for sediments was hence multiplied by the fraction of total chemical mass in the dissolved phase (i.e. in the sediment pore water), F_w. This fraction was calculated assuming equilibrium partitioning between sediment solids and pore water, using the fugacity capacities of sediment solids and pure water (calculated in BALTSEM-POP) and the basin specific volume fraction of sediment solids in sediments (given as input to BALTSEM-POP):

$$F_w = 1/(Z_{solidssed}/Z_w \times VF_{solidssed}/(1\text{-}VF_{solidssed})+1)$$

Case study: additional results

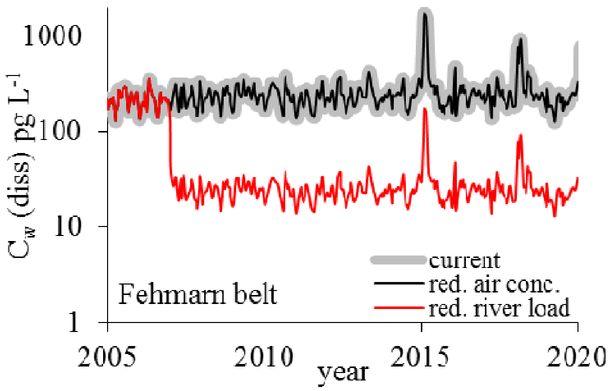

Figure S3. *Predicted surface water concentrations (10 m depth, dissolved in pg L^{-1} bulk water) in the Fehmarn Belt with emissions either constant at current levels, emissions to rivers reduced by 90% or air concentrations reduced by 90% (labelled current, red. river load and red. air conc. in the legend, respectively). Forcing scenario RWCL was used: The nutrient loads were fixed at a level representing the average between 1997 and 2003 (constant load, CL), and the climate scenario represents a random weather (RW) similar to today's conditions, i.e. no further global warming.*

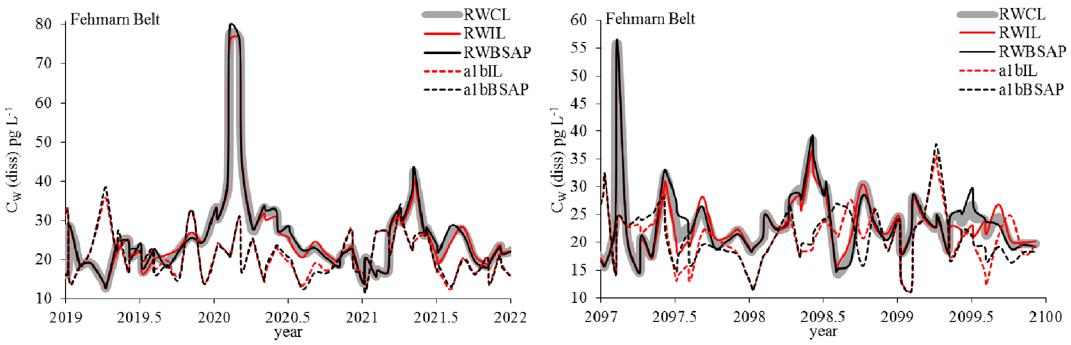

Figure S4. *Concentrations of freely dissolved D5 (C_W, pg L^{-1}) in surface water in the Fehmarn Belt during two time periods (2019-2022 and 2097-2100) calculated using five different scenarios for climate change and nutrient loads.*

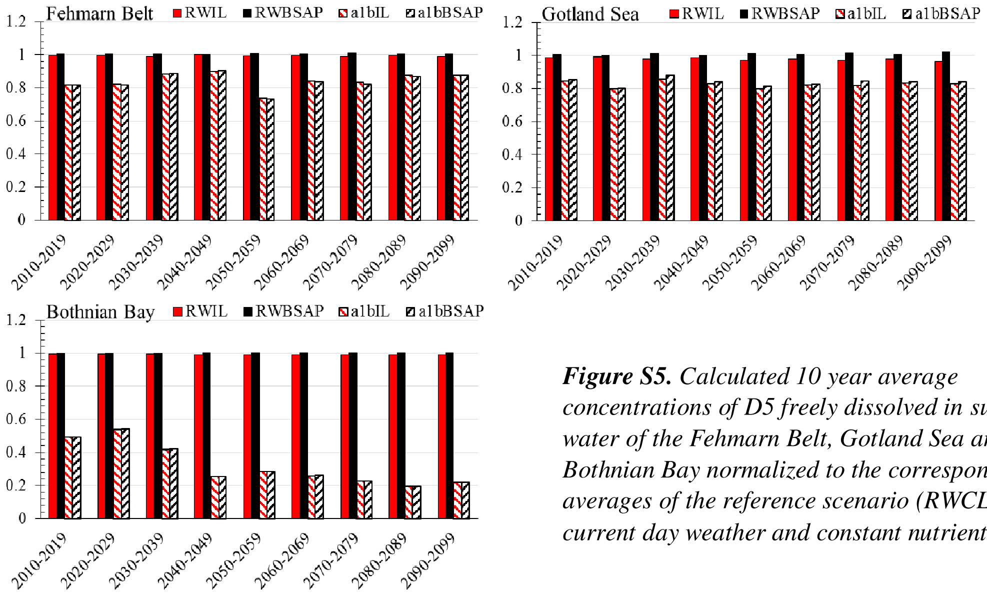

Figure S5. *Calculated 10 year average concentrations of D5 freely dissolved in surface water of the Fehmarn Belt, Gotland Sea and Bothnian Bay normalized to the corresponding averages of the reference scenario (RWCL, random current day weather and constant nutrient loads).*

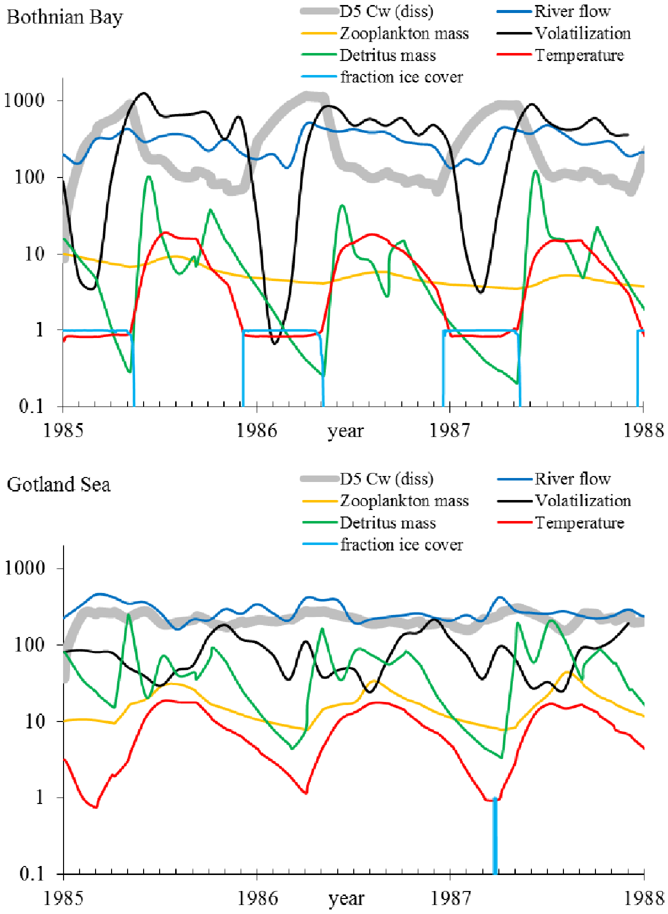

Figure S6. *Seasonality in concentration of D5 (dissolved) in surface water in the Bothnian Bay and Gotland Sea, and the processes/parameters mainly causing the variations. Note that absolute values are scaled to fit all data into the same graph, hence no units are displayed.*

References

Brooke, D. N., Crookes, M. J., Gray, D., Robertson, S., 2009. Environmental Risk Assessment Report: Decamethylcyclopentasiloxane; Environment Agency of England and Wales: Bristol. Available at: http://publications.environment-agency.gov.uk/pdf/SCHO0309BPQX-e-e.pdf

Gustafsson, B.G., Schenk, F., Blenckner, T., Eilola, K., Meier, H.M., Müller-Karulis, B., Neumann, T., Ruoho-Airola, T., Savchuk, O.P., Zorita, E., 2012. Reconstructing the Development of Baltic Sea Eutrophication 1850–2006. Ambio 41, 534–548.

HELCOM, 2013. Summary report on the development of revised Maximum Allowable Inputs (MAI) and updated Country Allocated Reduction Targets (CART) of the Baltic Sea Action Plan. Background document for the Copenhagen Ministerial Meeting 2013, 22pp. Available from www.helcom.fi.

Humborg, C., Mörth, C.-M., Sundbom, M., Wulff, F., 2007. Riverine transport of biogenic elements to the Baltic Sea- past and possible future perspectives. Hydrology and Earth System Sciences 11, 1593–1607.

Humborg, C., Mörth, C., Sundbom, M., Borg, H., Blenckner, T., Giesler, R., Ittekkot, V., 2010. CO2 supersaturation along the aquatic conduit in Swedish watersheds as constrained by terrestrial respiration, aquatic respiration and weathering. Global Change Biology 16, 1966-1978.

McLachlan, M.S., Kierkegaard, A., Hansen, K.M., van Egmond, R., Christensen, J.H., Skjoth, C.A., 2010. Concentrations and Fate of Decamethylcyclopentasiloxane (D-5) in the Atmosphere. Environmental Science & Technology 44, 5365-5370.

Meier, H.M., Höglund, A., Döscher, R., Andersson, H.C., Löptien, U., Kjellström, E., 2011. Quality assessment of atmospheric surface fields over the Baltic Sea from an ensemble of regional climate model simulations with respect to ocean dynamics. Oceanologia 53, 193–227.

Meier, H.M., Hordoir, R., Andersson, H.C., Dieterich, C., Eilola, K., Gustafsson, B.G., Höglund, A., Schimanke, S., 2012. Modeling the combined impact of changing climate and changing nutrient loads on the Baltic Sea environment in an ensemble of transient simulations for 1961–2099. Climate Dynamics.

Price, O.R., Williams, R.J., Zhang, Z., van Egmond, R., 2010. Modelling concentrations of decamethylcyclopentasiloxane in two UK rivers using LF2000-WQX. Environmental Pollution 158, 356-360.

Savchuk, O.P., Gustafsson, B.G., Müller-Karulis, B., 2012. BALTSEM a marine model for decision support within the Baltic Sea Region. Technical Report.

Whelan, M.J., 2013. Evaluating the fate and behaviour of cyclic volatile methyl siloxanes in two contrasting North American lakes using a multi-media model. Chemosphere 91, 1566-1576.

Xu, S., Kropscott, B., 2012a. Method for simultaneous determination of partition coefficients for cyclic volatile methylsiloxanes and dimethylsilanediol. Analytical chemistry 84, 1948-1955.

Xu, S., Kropscott, B., 2012b. Octanol/Air Partition Coefficients of Volatile Methylsiloxanes and Their Temperature Dependence. Journal of Chemical & Engineering Data 58, 136-142.
